# Efficacy and safety of conbercept as a primary treatment for choroidal neovascularization secondary to punctate inner choroidopathy

**DOI:** 10.1186/s12886-017-0481-8

**Published:** 2017-06-12

**Authors:** Yuting Peng, Xiongze Zhang, Lan Mi, Bing Liu, Chengguo Zuo, Miaoling Li, Feng Wen

**Affiliations:** State Key Laboratory of Ophthalmology, Zhongshan Ophthalmic Center, Sun Yat-sen University, 54 South Xianlie Road, Guangzhou, 510060 China

**Keywords:** Conbercept, KH902, Intravitreal injection, Choroidal neovascularization, Punctate inner choroidopathy, Efficacy

## Abstract

**Background:**

To evaluate the efficacy and safety of intravitreal conbercept (KH902) as the primary treatment of choroidal neovascularization secondary to punctate inner choroidopathy.

**Methods:**

This study was a retrospective, consecutive, observational case series. We reviewed medical records of 16 eyes (16 patients) with naive subfoveal or juxtafoveal choroidal neovascularization secondary to punctuate inner choroidopathy that were treated with intravitreal conbercept injections. All patients completed at least six months of follow-up. Best-corrected visual acuity (BCVA) was measured, and anatomical features were assessed with fluorescein angiography, indocyanine green angiography, and optical coherence tomography.

**Results:**

At the month-6 follow-up visit, best-corrected visual acuity improved from 0.70 ± 0.36 (with approximate Snellen equivalent of 20/100) to 0.44 ± 0.25 (20/50 in Snellen) logarithm of the minimum angle of resolution (logMAR) (*P* = 0.003). Mean improvement of vision was 2.6 lines, with 50% treated eyes (8 eyes of 16) showing an improvement of ≥3 lines and 62.5% (10 eyes of 16), obtaining an improvement of ≥2 lines; all 16 eyes had stable or improved vision. Mean central retinal thickness decreased from 294.94 ± 102.68 μm to 206.56 ± 61.71 μm (*P* = 0.005). Fifteen eyes (93.75%) showed absence of CNV leakage at the end of the study period. No conbercrept-related systemic or ocular adverse events were observed.

**Conclusion:**

Intravitreal injection of conbercept significantly improved visual and anatomical outcomes in choroidal neovascularization secondary to punctate inner choroidopathy over a 6-month follow-up period.

**Trial registration:**

ISRCTN85678307, retrospectively registered on May 11, 2017.

## Background

Choroidal neovascularization (CNV) is a disorder of the eye that impairs visual acuity and could lead to vision loss. CNV can develop secondary to punctate inner choroidopahty (PIC). In 1984, Watzke et al. [1] first described PIC, which is characterized by multifocal, small, yellow lesions, most likely located in the posterior pole, and that could lead to subsequent atrophy. PIC predominantly affects young myopic women and is not rare among the Chinese population [2]. Symptoms of PIC include blurred vision, photopsia, and scotomata. A recent study of PIC indicated that it is an inflammatory disorder [3]. The majority cases of PIC are self-limited with good visual prognosis. However, CNV developed secondary to PIC could lead to blindness [4].

The prevalence of CNV secondary to PIC in the Chinese population is high. CNV on average occurs in 22 to 69% of patients with PIC [4–6]; however, approximately 63% eyes with PIC develop CNV in Chinese patients [2]. A recent study found that PIC was associated with 50.4% of inflammatory CNV in a Chinese population [7]. Therefore, effective treatment of CNV is particularly critical for Chinese patients with PIC.

CNV is characterized by new blood vessel generation in the choriod layer of the eye, and it is a common cause of neovascular degenerative maculopathy. The molecular mechanism of CNV development is not well understood. However, studies have shown that vascular endothelium growth factor (VEGF) plays an essential role in the development of CNV. The first-line treatment of CNV is intravitreal injections of anti-VEGF agents administered on an as-needed basis. Currently available anti-VEGF agents include ranibizumab, bevacizumab and aflibercept [8–16].

Conbercept (KH902) is a fusion protein that consists of a human immunoglobulin Fc region and VEGF receptor key domains. It is similar in structure as aflibercept, an anti-VEGF agent that binds to all isoforms of VEGF-A, VEGF-B, and PIGF; however, conbercept has higher affinity to VEGF because of the addition of the fourth Ig-like domain of VEGFR-2 in the Fab fragment [17, 18]. Conbercept significantly improved visual acuity and anatomical outcomes in patients with polypoidal choroidal vasculopathy (PCV) or neovascular neovascular age-related macular degeneration (AMD) [18–20], and was approved to treat AMD by the State Food and Drug Administration of China in December 2013. This study aimed to evaluate the efficacy and safety of intravitreal injection of conbercept as the primary treatment of subfoveal or juxtafoveal CNV secondary to PIC.

## Methods

### Study design

This study was a retrospective, consecutive, observational case series. This study was approved by the medical ethics board of Zhongshan Ophthalmic Center, Sun Yat-sen University, and complied with the Declaration of Helsinki.

### Patients and study population

We reviewed the medical records of 16 eyes of 16 patients with CNV secondary to PIC. All patients met the inclusion and exclusion criteria. The patients were treated at Zhongshan Ophthalmic Center of Sun Yat-sen University between May 2015 and June 2016. All patients underwent intravitreal conbercept injections as the primary treatment for CNV secondary to PIC and completed at least 6 months of monthly follow-up examinations after the first treatment.

Patients with active subfoveal or juxatoveal CNV secondary to PIC were included in the study. If intraretinal edema or subretinal fluid was observed with optical coherence tomography (OCT) or leakage within lesion was observed with fluorescein angiography (FA), CNV was considered as active. PIC was defined as multiple, small yellow-white lesions or atrophy in the posterior pole that had typical manifestations on OCT [3]. And PIC lesions were around the CNV.

The following patients were excluded from this study: 1) CNV was secondary to other causes, such as AMD, PCV, fundus angioid streaks, trauma; 2) presence of any other ophthalmic diseases; 3) previous treatment of CNV, including intravitreal anti-VEGF drugs, photodynamic therapy, laser photocoagulation, or submacular surgery; 4) local or systemic corticosteroids or immunosuppressants or any intravitreal anti-VEGF drugs other than conbercept were given during the 6 months observational period; and 5) presence of systemic diseases or pregnancy.

### Intervention or observation procedure

At baseline, all patients had a complete ocular examination record. BCVA was assessed with the Snellen chart at 6 m. Intraocular pressure, and slit-lamp biomicroscopy, indirect ophthalmoscopy, fundus photography (Carl Zeiss, Inc., Jena, Germany), FA, indocyanine green angiography (ICGA) and OCT (Spectralis; Heidelberg Engineering, Dossenheim, Germany) results were reviewed.

At the first visit, all patients received a single 0.5 mg (0.05 ml) intravitreal injection conbercept (KH902) (Chengdu Kanghong Biotech Ltd., Sichuan, China). Additional injections were administered on follow-up visits if persistent or recurrent fluid involving the fovea was detected with OCT or FA.

### Main outcomes measure

BCVA was converted to the logMAR equivalent. Visual outcomes included the mean BCVA change in logMAR and the proportion of eyes whose BCVA improved (≥0.3 logMAR), were stable (within 0.3 logMAR), or decreased (≥0.3 logMAR) when compared to the baseline. A change of 0.1 logMAR was considered a change of one line. Anatomic outcomes, including CRT, subretinal or intraretinal fluid build-up, were recorded monthly, and angiographic leakage was assessed at the sixth visit.

### Statistic analysis

All data were expressed as mean and ± standard deviation (SD). The data were analyzed with the Statistical Package for the Social Sciences (SPSS Version 20.0, IBM Corporation, Chicago, Illinois, USA). Serial changes in BCVA and CRT were analyzed using wilcoxon signed-ranks test and paired t-test, respectively. Statistically significance was defined as the *P* value of less than 0.05.

## Results

### Baseline characteristics

A total of 16 patients were included in this study, the demographic details of 16 patients are shown in Table 1. One eye from each patient was treated. Out of the 16 treated eyes, 8 had subfoveal CNV, and 8 had juxtafoveal CNV. Briefly, the mean age of the patients was 48.44 ± 14.01 (range from 28 to 76); 13 (81.25%) of 16 were female. At baseline, the mean spherical equivalent refraction of treated eyes was −7.22 ± 4.42 diopters (range: 0 – −14 diopters), with 15 eyes (93.75%) being myopic. The mean BCVA, expressed in logMAR units, was 0.70 ± 0.36 (with approximate Snellen equivalent of 20/100); the mean CRT at baseline was 294.94 ± 102.68 μm.

**Table 1:** Demographic details of 16 patients who had intravitreal conbercept for choroidal neovascularization secondary to punctate inner choroidopathy

Patient No.	Gender /Age	Refractive diopter	Location of CNV	Best Corrected Visual Acuity		Central Retinal Thickness (μm)		Angiographic Leakage On FA		No. of Injection
				Baseline	Month 6	Baseline	Month 6	Baseline	Month 6	
1	F/45	-9	JF	20/200	20/80	256	186	YES	NO	2
2	F/54	-12	SF	20/80	20/50	303	203	YES	NO	1
3	F/31	-4.5	JF	20/160	20/50	207	196	YES	NO	1
4	F/48	-1	JF	20/40	20/40	176	155	YES	NO	1
5	M/66	-9	JF	20/32	20/50	205	295	YES	YES	3
6	F/39	-11.5	SF	20/100	20/32	305	213	YES	NO	2
7	F/48	-14	JF	20/100	20/63	204	150	YES	NO	1
8	F/60	-7.5	SF	20/333	20/80	310	202	YES	NO	3
9	M/28	-9.5	SF	20/50	20/40	457	275	YES	NO	1
10	F/29	-6.5	JF	20/32	20/32	199	194	YES	NO	1
11	F/53	-13	SF	20/400	20/250	326	303	YES	NO	1
12	F/53	-2	JF	20/50	20/40	185	180	YES	NO	1
13	F/76	-9	SF	20/333	20/160	484	301	YES	NO	1
14	M/65	-2	SF	20/50	20/40	428	165	YES	NO	3
15	F/45	0	JF	20/160	20/40	416	75	YES	NO	1
16	F/35	-5	SF	20/125	20/40	258	212	YES	NO	1

*CNV* choroidal neovascularization *F* female; *M* male; *SF* subfoveal; *JF* juxtafoveal

### Visual and anatomical outcomes

**Fig. 1:**
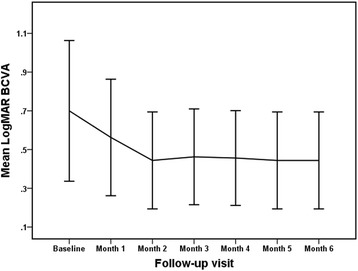
Mean best-corrected visual acuity (BCVA) (logMAR) at baseline and each monthly visit after commencement of intravitreal conbercept for the primary treatment of choroidal neovascularization secondary to punctate inner choroidopathy. *Error bar* represents standard deviation

BCVA of all patients was assessed with the Snellen chart at baseline and at subsequent monthly follow-up visit. The mean logMAR BCVA was 0.56 ± 0.30 (Snellen equivalent of 20/80) (*P* = 0.003) at the month-1 follow-up visit, 0.44 ± 0.25 (Snellen equivalent of 20/50) (*P* = 0.001) at the month-2 follow-up visit, 0.46 ± 0.25 (with approximate Snellen equivalent of 20/50) (*P* = 0.006) at the month-3 follow-up visit, 0.46 ± 0.24 (with approximate Snellen equivalent of 20/50) (*P* = 0.005) at the month-4 follow-up visit, 0.44 ± 0.25 (20/50 in Snellen equivalent) (*P* = 0.003) at the month-5 follow-up visit, and 0.44 ± 0.25 (Snellen equivalent of 20/50) (*P* = 0.003) at the month-6 follow-up visit; all values were significantly different when compared to the baseline BCVA (0.70 ± 0.36) (20/100 in Snellen equivalent) (Fig. 1). At the 6-month follow-up visit, no eye showed decreased BCVA; 8 eyes showed stable BCVA, and 8 improved BCVA. The mean improvement in visual acuity was 2.6 lines, with 8 eyes (50%) showing an improvement of ≥3 lines and 10 eyes (62.5%) an improvement of ≥2 lines; all 16 eyes (100%) had stable or improved vision.

**Fig. 2:**
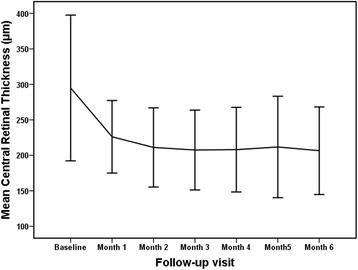
Mean Central Retinal Thickness (CRT) at baseline and each monthly visit after commencement of intravitreal conbercept for the primary treatment of choroidal neovascularization secondary to punctate inner choroidopathy. *Error bar* represents standard deviation

The mean CRT at the month-1, month-2, month-3, month-4, month-5 and month-6 follow-up visits were 226.13 ± 51.10 μm (*P* = 0.010), 211.19 ± 55.77 μm (*P* = 0.005), 207.50 ± 56.27 μm (*P* = 0.005), 208.00 ± 59.63 μm (*P* = 0.006), 211.81 ± 71.39 μm (*P* = 0.013), and 206.56 ± 61.71 μm (*P* = 0.005), respectively; all values were significantly different from CRT the at baseline level 294.94 ± 102.68 μm (Fig. 2). CRT decreased significantly at the end of the study period compared with baseline, with a mean decrease of 88.38 ± 108.44 μm.

**Fig. 3:**
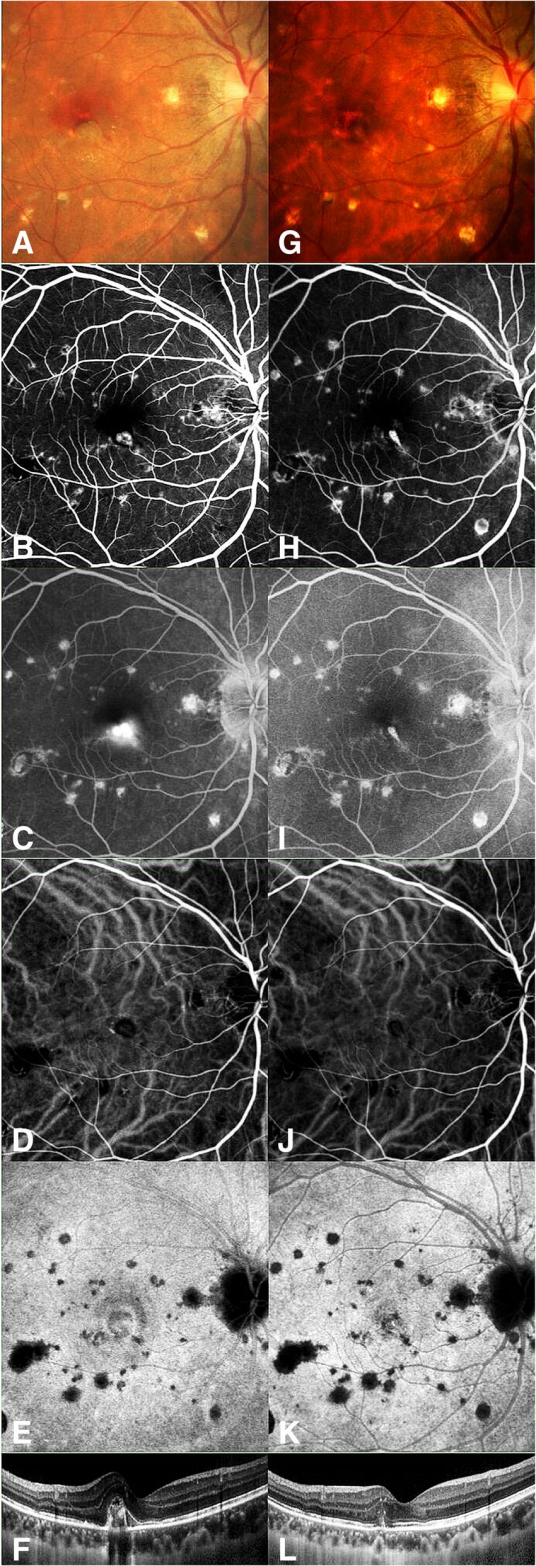
Color fundus photography, FA, ICGA and OCT images from the right eye of Patient 1 at baseline and month-6 follow-up visit. Fundus photo before treatment (**a**) demonstrated a *grey* juxtafoveal CNV with multiple small *yellowish-white* chorioretinal scars at the macula. The BCVA was 20/200. Early phase (**b**) and late phase (**c**) images of FA showed a juxtafoveal CNV with obviously leakage. Early phase (**d**) and late phase (**e**) images of ICGA showed a leaking CNV. OCT (**f**) demonstrated an elevated subretinal CNV with intraretinal edema and subretinal fluid involving the juxtafovea. Fundus photo (**g**) at sixth month after intravitreal conbercept treatment showed a scarring of CNV. The BCVA improved to 20/80. Early phase (**h**) and late phase (**i**) images of FA showed the reduced CNV lesion without leakage. Early phase (**j**) and late phase (**k**) images of ICGA showed that the CNV lesion turned to a small fibrovascular scar. Posttreatment OCT (**l**) at sixth month showed the reduction of CNV and the resolution of subretinal and intraretinal edema

At the last visit, subretinal or intraretinal fluid completely disappeared as assessed by OCT in 15 (93.75%) eyes (Fig. 3). Meanwhile, these 15 eyes had no CNV angiographic leakage on FA. All the 16 eyes had reduced or stable size of CNV at the last visit, and were without significantly change in number or size of PIC lesions on ICGA (Fig. 3). Patient 5 had recurrent fluid build-up involving the fovea and received reinjections at the third month and the fifth month follow-up visits. At the last visit, the eye of Patient 5 had significantly reduced CNV leakage but remained slight leakage on FA.

### Total numbers of treatments

On average, each treated eye received 1.50 ± 0.82 injections (a total of 24 injections were administered). Five eyes (31.25%) needed additional injections; 3 out of the 5 eyes required 3 injections in total, and the other 2 needed 2 injections. At the end of the study period, fifteen patients had CNV scarring and no recurrence of CNV.

### Complications

No systemic complications such as thromboembolic events or cerebral vascular events in this study were observed. Ocular complications such as intraocular inflammation, increase in IOP, cataract, endophthalmitis, and retinal detachment did not occur in the study.

## Discussion

CNV is a common complication of PIC [4, 6, 7, 21], and VEGF secreted by the retinal pigment epithelium plays an essential role in the development of CNV [22, 23]. Thus, anti-VEGF therapies have been used as a first line treatment of CNV, and the treatments have achieved favorable visual outcomes and are associated with few complications in treating CNV secondary to disorders such as exudative age-related macular degeneration (AMD) and pathologic myopia [24–26]. In addition, Kramer et al. reported that in patients with choroidal neovascularization related to inflammatory diseases, intravitreal injections of bevacizumab on an as-needed basis resolved leakage and subretinal fluid and improved visal acuity [27]. Several recent studies have reported that anti-VEGF agents, bevacizumab and ranibizumab were efficacious in treating CNV secondary to PIC [8, 28, 29]. Particularly, a case series study found that intravitreal anti-VEGF resolved CNV secondary to PIC over a 3–28 months follow-up period [10].

The present study evaluated the efficacy and safety of intravitreal injection of conbercept on an as-needed basis as the primary treatment for CNV secondary to PIC. Treatment with conbercept significantly improved visual acuity: the mean improvement was 2.6 lines, with 50% of treated eyes showing an improvement of ≥3 lines and 62.5% eyes showing an improvement of ≥2 lines. All of the treated eyes showed improved or stable BCVA. Treatment with conbercept also improved anatomical outcomes. CRT decreased significantly at the end of the study period compared with baseline, with a mean decrease of 88.38 (40.32–143.62) μm. Further, 15 eyes (93.75%) showed cicatricial CNV on FA; however, one treated eye showed signs of slight leakage at the end of the study period. These results indicate that conbercept has good efficacy in treating CNV secondary to PIC. In a prospective case series of 12 patients over 12 months, Zhang et al. [9] found that intravitreal injection of bevacizumab improved BCVA by ≥2 lines in 75% of treated eyes. In a retrospective case series, Mansour et al. [29] found that intravitreal injection of bevacizumab improved BCVA by 2.2 lines on average in treated PIC eyes with CNV. Cornish et al. [8] showed that BCVA of 11.1% treated eyes deteriorated one year after intravitreal bevacizumab or ranibizumab injection. In our study, no treated eyes showed a decrease in BCVA; however, because of the differences in study designs and other factors, the studies are not directly comparable. One conbercept-treated eye with persistent CNV experienced a relatively poor outcome. This patient with poor outcome after anti-VEGF monotherapy may be considered combination anti-VEGF and anti-inflammation therapy. This patient with relatively poor outcome after anti-VEGF monotherapy may be considered combination anti-VEGF and anti-inflammation therapy. Although this is not a prospective study, and is not a comparative trial, these results indicate that conbercept is effective in treating CNV secondary to PIC.

## Conclusions

Our results indicated that intravitreal conberept is efficacious and safe as a primary treatment for CNV secondary to PIC over a 6-month follow-up period. Intravitreal conbercept could be considered as a treatment option for CNV secondary to PIC. Further multi-center, randomized, long-term, and controlled studies are warranted to determine fully the efficacy and safety of intravitreal conbercept in the treatment of CNV secondary to PIC.

## Abbreviations

BCVA: Best corrected visual acuity
CNV: Choroidal neovascularization
CRT: Central retinal thickness (CRT)
EDTRS: Early Treatment Diabetic Retinopathy Study
FA: Fluorescein angiography
ICGA: Indocyanine green angiography
LogMAR: Logarithm of the minimum angle of resolution
OCT: Optical coherence tomography
PIC: Punctate inner choroidopathy
VEGF: Vascular endothelial growth factor
